# Calcium Phosphate Bone Cements Including Sugar Surfactants: Part Two—Injectability, Adhesive Properties and Biocompatibility

**DOI:** 10.3390/ma3125111

**Published:** 2010-12-02

**Authors:** Ariane Bercier, Stéphane Gonçalves, Helène Autefage, Fabienne Briand-Mesange, Olivier Lignon, Juliette Fitremann

**Affiliations:** 1 Université de Toulouse, Laboratoire des IMRCP, CNRS-Université Paul Sabatier, Bâtiment 2R1, 118 Route de Narbonne, 31062 Toulouse Cedex 9, France; 2 Teknimed SA, 11 rue Apollo, ZI Montredon, 31240 L'UNION, France; 3 Unité INSERM 563, CPTP, Dpt. LML, Bâtiment C, Hôpital de Purpan, BP3028, 31024 Toulouse cedex 3, France

**Keywords:** calcium phosphate, surfactant, sugar, injectability, adhesiveness, sticky

## Abstract

Addition of sugar surfactants, sucrose fatty acid esters and alkylpolyglucosides to a calcium phosphate cement, designed for bone reconstruction, is described. Thanks to their adsorption at the surface of the calcium phosphate particles, the sugar surfactants allowed a full injectability and brought a very good workability. Injectability was measured by monitoring force-distance curves. With some of the selected sugar surfactants adhesive properties of the cement pastes were also observed, which were measured by tack tests. Finally, some properties related to biological applications are described, including gentamicine release and osteoblast viability experiments. The whole study demonstrates that addition of these mild surfactants improved several properties of the calcium phosphate cement, without impairing function.

## 1. Introduction

Calcium phosphate bone cements are now well recognized materials for their usefulness in surgical practice for bone reconstruction [1]. Interest in these materials is due to the following properties. First, their mineral composition tends to be very close to the mineral composition of bone itself: after setting and mineral transformation, cements transform in hydroxyapatite, which make them excellent biocompatible materials. Secondly, they can be prepared as a paste, so that surgeons can precisely fill bone defects, before hardening *in situ*. Thirdly, calcium phosphate cements are osteoconducting materials, and tend to resorb to the benefit of true bone, but the quality and extent of cell colonization and bioresorption depends greatly on the porosity. However, the main drawback is their lack of mechanical resistance, making them only useful for bones not subjected to high loads.

While several formulations are offered on the market, some properties need to be improved and drawbacks need to be resolved [2,3]. One desirable property for these cements made *in situ* is injectability. If the cement paste can be injected through a cannula, it is the least invasive surgical technique. As recently reviewed [4,5], inorganic additives or polymers can be added to improve injectability. Polymers are especially used to increase the viscosity of the aqueous phase, thus decreasing the filter pressing phenomenon. However, one kind of additive—surfactants—has been quite poorly studied in this field. Yet, surfactants are known to adsorb at liquid-liquid interfaces (typically water—oil emulsions), liquid-gas interfaces (foams) and liquid-solid interfaces (particle dispersions). Since calcium phosphate cements are concentrated dispersions of particles in an aqueous solution, addition of a correctly selected surfactant should improve the stability of the formulation, change the rheological properties and bring a lubricating effect [6]. To our knowledge, addition of surfactants to biomedical calcium phosphate cement pastes has only been described in a few studies. In these studies, it has been shown that addition of surfactant increased the porosity, through the stabilization of air bubbles in the cement (foaming properties). Otherwise, improvement in injectability was not clear, except recently with albumen proteins and sorbitan esters. However, in the first example, quite high amounts of albumen (10 wt % of the calcium phosphate powder) were required for reaching complete injection [7], and in the second example, full injectability could not be achieved [8].

In this context, our interest is focused on the addition of sugar surfactants, and more especially, sucrose esters surfactants, to calcium phosphate cement, taking advantage of our good knowledge of these molecules [9]. Sucrose esters (SE) are prepared by trans-esterification of fatty acid esters with sucrose. This reaction gives surfactants that split in sugars and fatty acids upon hydrolysis. Today they are used as food additives (E 473) or in cosmetic and oral or topical pharmaceutical formulations. Our former experience on the stabilization of solid dispersions by these surfactants led us to expect quite good results on the texture and rheology of calcium phosphate dispersions too [10,11]. Various hydrophile-lipophile balances are available depending on the fatty acid chain length and the number of chains grafted on sucrose. Thus, we tuned this parameter and showed its effect on different properties of the cements. Compared with other surface-active agents already studied, they are non-ionic, non-irritating, and they are synthetic products ensuring a good reproducibility. The sucrose esters used in this study are: sucrose laurate (SE16L, mainly monolaurate), sucrose palmitate (SE16P, mainly monopalmitate), sucrose stearate (SE11S, mixture of mono, di, tri-stearate), sucrose stearate (SE5S, mainly high substitution degree, hydrophobic). In addition, sugar surfactants from the family of alkylpolyglucosides (APG) have been tested: Montanov 68EC (M68EC, mixture of cetylalcohol and cetylglucoside), Montanov 14 (M14, mixture of myristylalcohol and myristylglucoside) and Oramix NS10 (ONS10, decylglucoside). This family is more represented than sucrose esters in terms of production levels, and also displays good toxicological profiles [12]. Chemical structures of these surfactants are given in the accompanying Supplemental Materials.

In a first part (Part One [13]), we have shown how the addition of these sugar surfactants (sucrose esters, alkylpolyglucosides) to calcium phosphate powder led to a strong improvement in porosity and texture, while keeping a correct setting time and a good cohesion of the paste. In this part, we first focused on the effect of sugar surfactants on the injectability of the pastes. Different methods have been used in the literature for measuring injectability quantitatively. A first method is based on the weight percent of paste extruded by hand from a syringe. However, with this method, it is not possible to differentiate between two cement pastes that both led to full extrusion, even if the operator can clearly feel by hand that the extrusion is more or less difficult. Then, to get data about the extrusion force, in some studies, mechanical test machines have been used to measure the maximal applied force [7,14,15,16] or to monitor the force applied during the whole extrusion [5,17,3]. In other studies, the pressure increase during extrusion by hand has been measured [18]. In our study, injectability has been monitored by recording the force-displacement curves during cement paste extrusion, using a device similar to the one described by Bohner *et al*. [3].

During our work, another effect specifically due to the addition of the sugar surfactants has been observed. At the macroscopic scale, sugar surfactants in more or less concentrated aqueous medium display viscoelastic properties. At the molecular scale, they display numerous hydroxyl groups available for hydrogen bonding. Both properties resulted in the development of adhesive properties. The phenomenon is well-known for some typical carbohydrates, such as cellulose derivatives or starch gels, and has also been studied for sugar and many other carbohydrates, in the context of food products. Thus, we noticed that the cement pastes obtained after addition of some sucrose surfactants were very sticky. A quantitative measurement of this property can be obtained by performing probe-tack measurements on the cement pastes. In the context of surgical applications, this property could be useful during the filling of bone defects by ensuring a better contact with bone, better positional stability of the cement *in situ*, and easier application of the paste. For example, the adhesive properties of acrylic bone cements [19] or calcium phosphate cements [20] have been studied in the context of the use of cement as the interface between metal prosthesis and the bone. A good adhesiveness should improve the long-term fixation of the prosthesis. For this reason, in our study, we selected polymers, but also bone and stainless-steel surfaces for performing tack-tests.

Finally, some biological properties have been studied. Addition of biologically active molecules, such as antibiotics [21,22,23,24,25,14], anti-tumor or growth factors [26,27,28] to bone cements has already been described and reviewed recently [29]. Addition of antibiotics aims to avoid infectious complications related to bone surgery, while anti-tumor agents are used with the purpose of ensuring a local and slow delivery of the drug. In our study, the *in vitro* release of an antibiotic, gentamicine, added to the cement is described. Otherwise, osteoblasts have been cultured on cement tablets with increasing porosity including sucrose esters or alkylpolyglucosides. These experiments gave a first estimation of the viability of osteoblasts in contact with cements loaded with these surfactants, and also the degree of penetration of the cells inside. 

## 2. Results and Discussion

### 2.1. Injectability

Addition of sucrose esters to the cements brought a very significant improvement of workability and injectability. As expected, by hand, injectability of Cementek alone is quite poor (44% of mass extruded), since it has not been designed to be injectable, while injectability of Cementek LV, which contains an additional 1% polydimethylsiloxane to help injectability, is good (84%; see Table 1). Addition of sucrose esters led to a strong improvement in the injectability of both Cementek and Cementek LV, allowing the complete and easy extrusion of the paste (the 5–6% missing up to 100% correspond to the cement paste remaining in the needle that cannot be extruded). From Table 1, two effects can be extracted. First, increasing the amount of surfactant over 1% generally does not further improve the injectability: 1% is enough, and probably less than 1% can bring the effect. Indeed, very little surfactant is usually needed for improving the quality of solid-liquid dispersions, corresponding to a full coverage of the surface of the particles. Increasing the amount of surfactant over 1% tends to decrease the amount of cement that can be extruded by hand. This is due to viscosity effects induced by the higher amount of surfactant. Secondly, on the whole, better results were obtained with sucrose esters compared to alkylpolyglucosides. 

**Table 1:** Injectability of the Cementek and Cementek LV cement pastes (weight percent of cement extruded by hand).

	surfactant	0%	1%	2%	3%	5%
Cementek	SE11S	44	91	88	86	71
	SE16P		95	94	92	86
	SE16L		90	94	88	95
	M68EC		85	-	86	69
	M14		67	-	86	83
	ONS10		93	-	85	81
Cementek LV	SE11S	84	-	91	-	-
	SE16P		-	87	-	-
	M68EC		-	70	-	-
	M14		-	71	-	-

**Figure 1:**
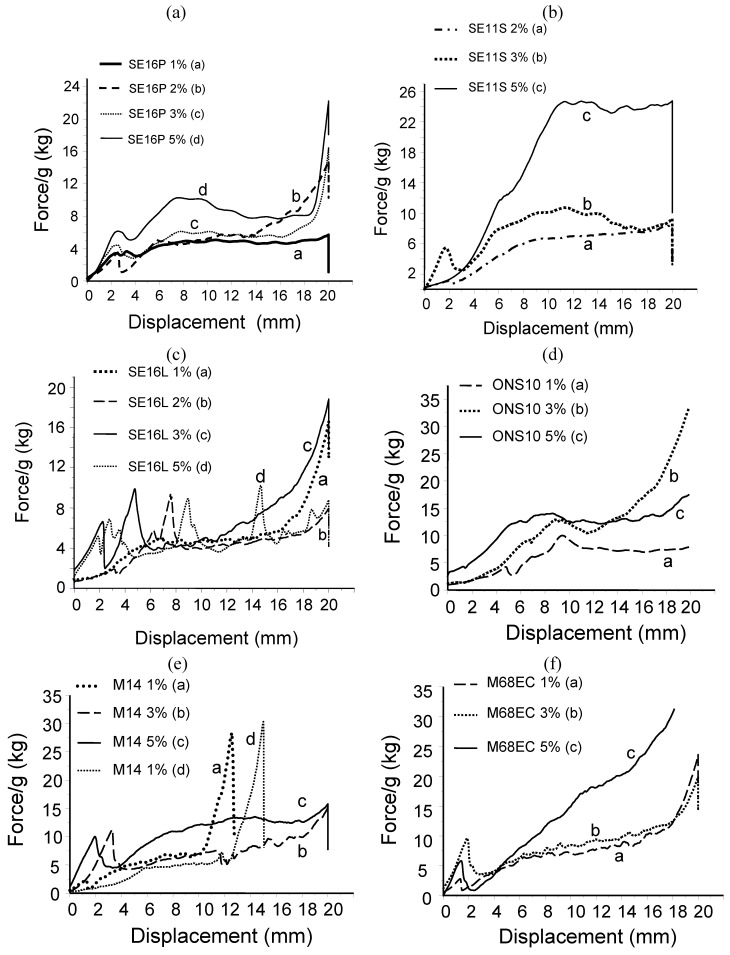

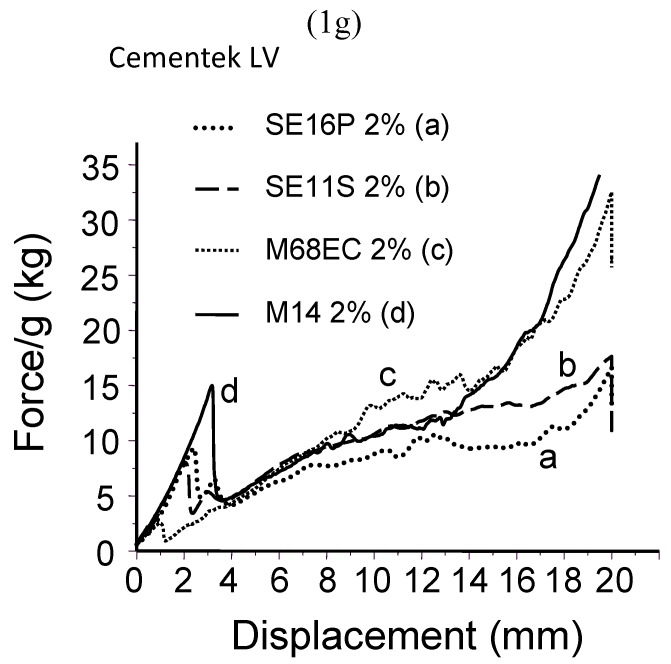
Injectability of Cementek with: (**a**) SE16P ; (**b**) SE11S; (**c**) SE16L; (**d**) ONS10; (**e**) M14; (**f**) M68EC; (**g**) Injectability of Cementek LV with the different surfactants at 2%.

More complete data were extracted from force-displacement curves measured during extrusion (Figure 1a–g). We found quite good reproducibility, significant and quantitative results with the mechanical test machine. The curves obtained are quite similar to those obtained by Bohner *et al.* [3] or Gauthier *et al* [17]. The first ones tested the injectability of cement added with xanthan gum, while the second ones tested a formulation of calcium phosphate ceramic granules with hydroxypropyl-methyl-cellulose. In our study, in the case of cement without additives, the mechanical tester reached very quickly force overload and stopped. In this case, demixing and packing is observed and the curves cannot be registered. Conversely, by adding sugar surfactants, complete extrusion can be monitored with nearly any surfactant, independent of the amount. Many curves displayed the same pattern (with Cementek: Figure 1a–f or with Cementek LV: Figure 1g). Most curves displayed a small peak or irregularities at the onset, which has been first attributed to a step corresponding to the packing of the paste, loosely introduced in the syringe, and secondly to a slightly higher force needed for pushing the very beginning of the paste in the needle. Once the flow is established, this starting peak decreased. Most of the curves also displayed a plateau or a very slightly increasing force during a large part of the extrusion process. This means that in these samples, the viscosity does not change a lot and the paste keeps a good homogeneity. This plateau, expressed in kg, is also significant of a more or less easy extrusion, and depends on the additive and its amount. Many of the additives enabled a full extrusion at a force lower than 10 kg, namely, affordable by hand. Up-scaling with a full sample of Cementek (16 cm^3^, with SE16P) injected in the surgical device (11G needle) also demonstrated a very easy extrusion by hand. On few other compositions (with Cementek: M68EC 5%, ONS 3%, SE16L 3%; with Cementek LV Figure 1g: M68EC 2% and M14 2%), this behavior with constant or slow increase of the force is not observed. Instead, a strong increase of the force is observed for the entire extrusion. Finally, with SE11S 5%, after a rapid increase of the force, a plateau at a higher force (≈25 kg) is observed at the end of extrusion. As it was found during extrusion by hand (Table 1), the curves also showed that over 1% the injectability was less easy. In Table 1, this is shown by the lower quantity of the extruded mass, while in the curves in Figure 1, this is shown by the higher pressure required for extrusion. With the machine, however, most of the time complete injectability is still possible, because a higher force can be applied compared with by hand. In fact, the stabilization of the solid-liquid dispersion, which impairs demixing, and the lubrication effect requires a low amount of surfactant. Any higher percentage of surfactant is effective also for stabilization and lubrication, but induces an increase of the viscosity. Classification of injectability at 1% surfactant with Cementek can be made in the following order: SE16L (4 kg) ≈ SE16P (4 kg) > ONS10 (7 kg) ≈ M68EC (7 kg) > M14 (blocking before the end). With Cementek LV loaded with 2% surfactant, this classification still holds, but on the whole, higher forces were required for extrusion compared with Cementek loaded with 2% or even 3% surfactant. Combination of polydimethylsiloxane and sugar surfactant as lubricating agents thus did not bring a favorable synergy. Finally formulations with Cementek loaded with 5% ONS10 and 5% M14 showed an easiest extrusion than with 5% SE11S or 5% M68EC. Alkylpolyglucosides (APG), namely M68EC, M14 and ONS10, led to full injectability too, except for M14 1%. However, most often, higher forces were required compared with sucrose esters. In conclusion, the sucrose ester SE16P showed the best behavior in terms of injectability, and any amount from 1 to 3% can be selected since nearly the same force for extrusion was required. These experiments also demonstrated that the force required for extrusion is quite sensitive to the precise structure of the selected surfactants. Despite that their structures are quite similar (see Supplemental Materials), significant differences have been obtained. This could explain why an improvement of injectability was not pointed out in former studies in which surfactants were added (SDS, Sorbitan esters [30,8]), or why in other studies, much more additive was required to get full injectability (albumen [7]).

### 2.2. Probe-tack adhesion measurements

During the preparation of the cement pastes, we visually observed quite clearly that some of them developed a sticky behavior before setting. This behavior depended on the sugar derivative included. Probe-tack measurements appeared the most appropriate to get a quantification of these observations. In these measurements, a probe (here, a cylinder), is brought in close contact with the cement paste during a fixed time (20 s) at a fixed force (25 kPa), then removed at a fixed and low rate (separation rate: 0.2 mm/s; see scheme in Supplemental Materials). These parameters were selected according to usual values for this kind of experiment and after few adjustments, until obtaining the best adhesion curves with the highest values of adhesion forces and energies. The resulting force-displacement curves were monitored. On the basis of these curves, two values featuring the adhesiveness are determined: the maximum force (N) corresponding to the peak of the curve and the adhesion energy (J/m^2^) obtained from the value of the area under the curve. The maximum force can be normalized by dividing by the probe surface, giving the maximum tensile stress (mN/mm^2^, kPa). In the case of our cement pastes, we selected a cylinder probe made of various materials, including bone, plastic (nylon) and stainless steel. All of them were polished. It must be pointed out that the measurements were probe-dependent, and the results must be considered by comparison to each other with the same probe.

**Figure 2:**
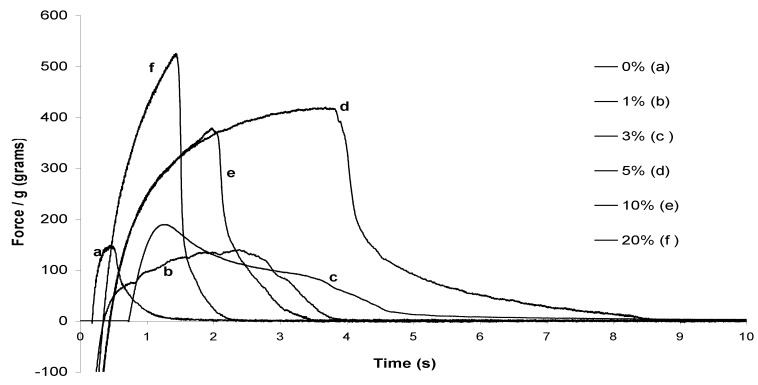
Probe-tack adhesion curves of Cementek with increasing amounts of SE16P (nylon probe).

**Figure 3:**
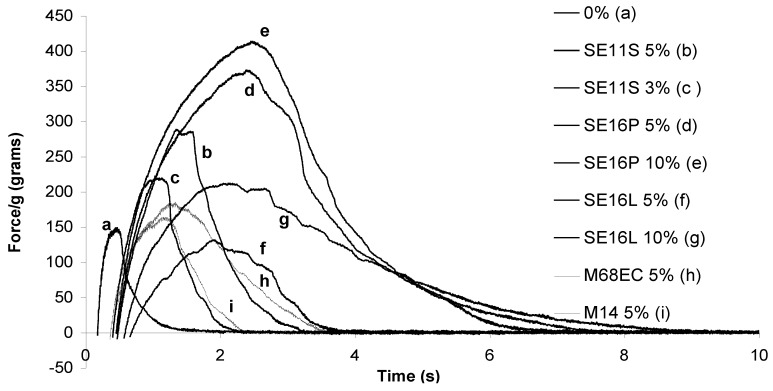
Probe-tack adhesion curves of Cementek with different surfactants (bone probe).

A first series of experiments made with a nylon probe showed that SE16P displayed the highest adhesive properties (Table 2). In the case of SE16P, a good increase of stickiness was observed with as little as 1% additive, compared with the cement without additive. However, the adhesiveness reached a maximum for 5% of SE16P, then decreased with a higher amount of surfactant (Figure 2 and Table 2). Sucrose esters with higher hydrophilicity (SE16L) or higher hydrophobicity (SE11S, SE5S) were less sticky. The tack tests were also made with a probe made of bone. Sucrose esters and alkylpolyglucosides were tested as additives at 3, 5 or 10% in the cement. The resulting curves are reported in Figure 3, and the corresponding normalized adhesion energies and maximum stresses are reported in Table 3. With bone, the addition of sugar surfactant again increased the stickiness and in this case SE16P also displayed much higher adhesive behavior than the other surfactants. At 5% surfactant, either with nylon or bone, adhesiveness can be ranked in the following order: SE16P >> SE16L, SE11S, SE5S, M68EC, M14 >> ONS10. The higher sticky behavior of SE16P was also observed independent of the amount of surfactant. Similar results were obtained for Cementek LV with the different surfactants. Tests with stainless steel as the material coming in contact with the cement pastes have also been done (see Supplemental Materials). The adhesion energies were much lower compared with the use of nylon or bone, but the tendency of SE16P to stick more was observed again. Some qualitative tests were made by adding non-amphiphilic monosaccharides, disaccharides, oligosaccharides or polysaccharides in the cement. None of them led to an increase in the sticky properties, showing the role of the viscoelastic properties of sugar surfactants on adhesive properties. 

**Table 2:** Adhesion energy to nylon and maximum tensile stress of cement pastes with the different sugar surfactants.

Sugar surfactant	Surfactant amount (%)	Adhesion Energy (J/m^2^ )	Maximum Tensile Stress (mN/mm^2^, kPa)
none	0	0.6	3.4
SE16P	1	2.0	4.4
SE16P	3	2.4	5.8
SE16P	5	9.3	13.1
SE16P	10	3.4	11.9
SE16P	20	2.8	16.5
SE16L	5	1.7	6.7
SE11S	3	2.3	4.3
SE11S	5	0.5	3.4
SE5S	1	0.9	5.3
SE5S	3	1.6	5.0
SE5S	5	1.4	5.1
SE5S	10	1.8	8.6
M68EC	5	1.4	5.8
M14	5	0.9	3.8
ONS10	5	0.08	1.2

**Table 3:** Adhesion energy to bone of cement pastes with the different sugar surfactants.

Sugar Surfactant	Surfactant amount (%)	Adhesion Energy to Bone (J/m^2 ^)	Maximum Tensile Stress (mN/mm^2^, kPa)
none	0	0.4	4.7
SE16P	5	6.5	11.7
SE16P	10	7.4	12.9
SE16L	5	1.4	4.1
SE16L	10	4.6	6.6
SE11S	3	1.2	6.9
SE11S	5	2.2	9.0
M68EC	5	1.9	5.8
M14	5	1.1	5.2
ONS10	5	0.01	0.5

The order of magnitude of the adhesion energy and maximum tensile stress is similar to what has been obtained in the case of solutions of some carbohydrates, such as maltodextrins (maximum stress ≈ 1–3 kPa) [31], carrageenan (maximum stress ≈ 10–20 kPa) [32] or sugar glasses (maximum stress ≈ 1–3 kPa) [33]. We can notice also that the higher values of adhesiveness obtained with sucrose esters did not require such high amounts, since 5% of additive was enough to get these values. They remain, however, far from the values corresponding to “true” adhesives. By taking the example of acrylic pressure-sensitive adhesives, the order of magnitude of maximum tensile stress lies between ≈100–1000 kPa [34]. Another example can be given: a stick of paper white glue has been used as a reference with our probes in our conditions, for which adhesion energy was found to be about 18 J/m^2^ on nylon (max. stress 14 mN/mm^2^), and 13 J/m^2^ on bone. As a consequence, the adhesive properties of these cements should be helpful for assisting a better positioning of the cement and to ensure a better contact with bone, but without impairing easy handling or injectability by being too sticky.

### 2.3. Gentamicine release

**Figure 4:**
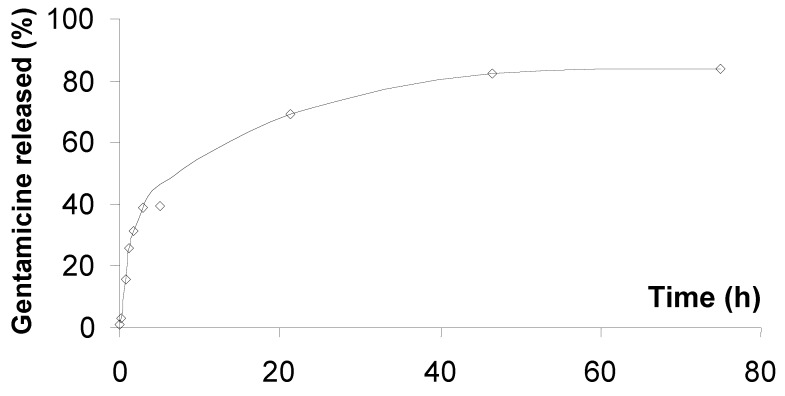
Gentamicine release from cement containing 2% SE16P.

Tests of controlled release of gentamicine included in cement have been made. Gentamicine (loaded at 25.15 mg/g of cement powder, corresponding to 2.5% w/w of cement powder or 1.7% w/w of solid + liquid phase) was mixed with the cement powder containing 2% SE16P. After mixing with the aqueous phase, the resulting cement paste was poured in a controlled volume of water (6 mL). Aliquots were sampled with time and the amount of gentamicine released was quantified by HPLC. The resulting curve is plotted in Figure 4. It can be seen that the release remained progressive, and quite slow without “flash” effect. After three days, 84% of the antibiotic was released in the solution. For 1 g of cement powder, 72% was released the first day, corresponding to 18.9 mg over 24 h. The second day, 10% more was released (2.5 mg). Finally the third day, 2% more was released, corresponding to 0.50 mg over this period. A slow continuation of the release is expected over this three day period. The kinetics of release at the beginning of the curve was quite similar to what has been observed by other authors [21]. It was somewhat faster compared with tetracycline incorporated in the same cement (Cementek, 5% tetracycline added), but without additive [14], which could be explained by both the higher porosity of Cementek with 2% SE16P added and the much higher hydrosolubility of gentamicine. To compare with implants for which *in vivo* release has been studied, a calcium phosphate glass implant loaded with gentamicine (7 mg gentamicine for 200 mg of CaP glass, namely 35 mg/g) led to a continuous increase of the gentamicine concentration in the surrounding bone during four weeks, with a maximal concentration of 14 μg/mL reached [35]. In another study, a calcium phosphate implant coated with PLA and loaded with 3.5% w/w gentamicine reached a maximal concentration of gentamicine of ≈18 μg/g of bone after one week, while the therapeutic range of gentamicine is 3–10 μg/mL [36].

### 2.4. Osteoblast viability and morphology

**Figure 5:**
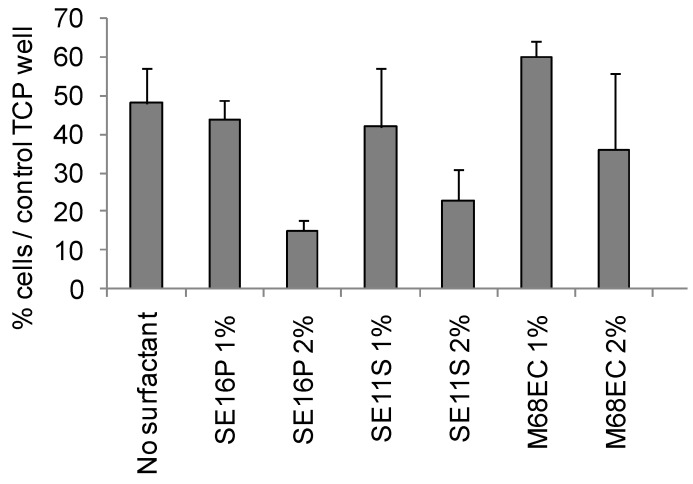
Osteoblast viability measured by MTT assay as a function of surfactant concentration.

**Figure 6:**
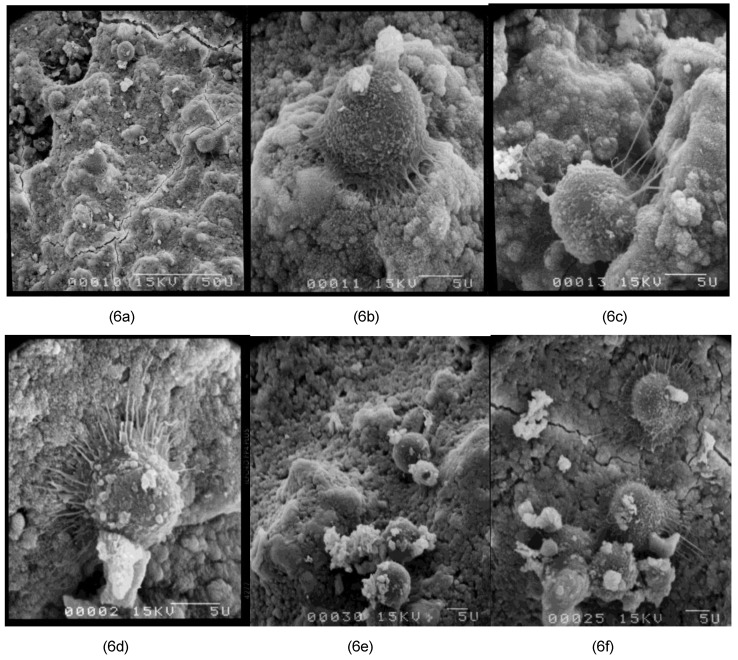
Morphology of osteoblasts at 24 h at the surface of cement tablets. Bar = 5 microns, except for 6a, 50 microns. (**a**,**b**,**c**) SE16P; (**d**) SE11S; (**e**) M68EC; (**f**) No surfactant.

Human osteoblasts were seeded on cement tablets containing 1 or 2% of sucrose esters or alkylpolyglucosides, in order to test the biocompatibility of the samples. After 24 hours of culture on the cements, the percentage of viable cells, relative to the tissue culture plastic wells (control TCP well), was determined by MTT (Figure 5) and the morphology of the cells was observed by Scanning Electron Microscopy (SEM) on selected compositions (Figures 6a–f). The following trends can be extracted from the results. By adding 1% of sucrose esters (SE16P or SE11S), the number of viable cells was similar to the tablet without surfactant. This number of viable cells was a little higher when 1% M68EC was added compared to the tablet without surfactant. When 2% of surfactant was added, a systematic decrease of the cell number was obtained. In some studies with sintered calcium phosphate cements, decrease of the cell number with increasing porosity has been observed [37] (or not [38]). This effect on cell viability has been attributed to the cytotoxicity of the released particles [39,40,41]. Such an effect can be an explanation for the decrease observed in our compositions from 1 to 2% of surfactant, since hydraulic cements are even more prone to particles release. Morphology and spreading of the osteoblasts cultured for 24 h on the tablets were observed by SEM (Figures 6a–f). The SEM pictures tended to show strong anchorage of osteoblasts cultured on cement tablets with sucrose esters. Cells were spherical and exhibited numerous and sometimes very long filopodia. This spherical morphology corresponds to the starting phase of the proliferation [42]. Sections of tablets have also been observed in order to assess the penetration depth of the cells inside the tablets. Osteoblasts could be seen up to two-thirds of the thickness of the tablet, showing a penetration and a survival of the cells inside the sample over more than 1 mm after 24 h, through interconnected cavities. With the alkylpolyglucoside M68EC, cells looked completely spherical, and filopodia were not clearly observed. The cells spread over about one-third of the sample thickness. In the sample without surfactant, cells were seen essentially at the surface of the tablet. These results are correctly in relation with the measured porosity, decreasing from sucrose esters to M68EC to samples without surfactant (respectively, 54%, 53%, 51% and 46% for porosity with SE11S, SE16P, M68EC and without surfactant, see Part One [13]). 

## 3. Experimental Section

### 3.1. Materials

Calcium phosphate cements were prepared from the ready-to-use "kit" Cementek and Cementek LV (Teknimed, Toulouse, France). Cementek is composed of a calcium phosphate powder (38% α-tricalcium phosphate, 49% tetracalcium phosphate and 13% sodium glycerophosphate) and an aqueous solution (13.8% phosphoric acid and 3.4% calcium hydroxide in water). In Cementek LV, the composition of the calcium phosphate powder is 38% alpha-tricalcium phosphate, 49% tetracalcium phosphate, 12% sodium glycerophosphate and 1% polydimethylsiloxane.

Food grade sucrose esters (sucrose laurate -SE16L-, sucrose palmitate -SE16P-, sucrose stearate-SE11S, medium substitution degree-, sucrose stearate -SE5S, high substitution degree, hydrophobic-) were kindly supplied by Stearinerie Dubois (France). Alkyl-polyglucosides (Montanov 68EC-M68EC, mixture of cetylalcohol and cetylglucoside, Montanov 14-M14, mixture of myristylalcohol and myristylglucoside-, Oramix NS10 -ONS10, decylglucoside-) were kindly supplied by Seppic (France). 

### 3.2. Sample preparation

The desired amount of sugar surfactant was added to the calcium phosphate powder, and the powders crushed together in a mortar for two minutes. The powder mixture was transferred into the plastic bowl provided in the kit, and the aqueous calcium phosphate solution was added, according to the proportions given by the manufacturer. More precisely, samples were made from 2.00 g of powder and 0.86 g of solution. The proportion of sugar surfactant added was defined as the weight percent of the whole mixture powder + liquid, and the mass of surfactant was neglected in the calculation. Typically, cement with 1% of sugar surfactant is composed of 2.00 g powder, 0.86 g solution and 29 mg surfactant. After addition of the liquid, the mixture was mixed for three minutes with a small flat spatula during which a smooth paste was formed. After this delay, the paste was used for various experiments. For all experiments, the time t = 0 corresponded to the mixing of the cement with the acidic solution. The powders were sterilized by gamma irradiation after mixing with additives and the liquid phase was also sterilized by gamma irradiation before use.

### 3.3. Injectability

Injectability was assessed by two methods. A first method consisted in pouring the paste into a plastic syringe (2 mL, 1.05 cm external diameter, with screw lock), immediately after the three minutes of mixing. The paste was then extruded quickly, at 5 min 30 s after the mixture of powder-solution had started, through a stainless-steel needle of 46 mm length and 2.03 mm external diameter (14 G). The needle was chosen with a ratio internal diameter/length downscaled compared to the one used in surgical devices (125 mm × 11 G), by using the Poiseuille’s Law as a very coarse approximation. The mass extruded was measured and expressed in percent compared to the initial total mass. 

A more quantitative method has been also set up, for monitoring the force needed during the extrusion of the cement. For this purpose, a home-made system was built. The injection device was adapted in order to fit into a Mechanical Test Machine (Texture Analyzer TA XT2 from Stable MicroSystems), and Force–Displacement curves were monitored. From a regular plastic syringe with screw lock (2 mL, 1.05 cm external diameter), the plastic piston was replaced by a stainless-steel one in order to resist pressure and was screwed to the arm of the mechanical tester. On the other side, the plastic cylinder of the syringe was fitted into a stainless-steel cylinder with 1.05 cm internal diameter, put on the machine basement but not fixed (see Supplemental Materials: “Home-made devices”). The freshly prepared cement paste was poured in the syringe, then extruded 5 min 30 s after the mixture of powder-solution had started. The force was monitored along a 20 mm displacement course of the piston.

### 3.4. Probe-Tack Measurements

After mixing the pastes containing some of the sugar surfactants, quite strong adhesive behavior was observed visually. This has been quantified by tack tests, by using a home-made device, at room temperature. For this purpose, a mobile steel cylinder (20 mm diameter) was secured to the arm of the Texture Analyzer. At the end of this cylinder, a cylindrical tablet (12 mm height, surface S = 314 mm^2^) made of various materials was fit. The materials tested were: nylon, bone (from bovine tibia) and stainless steel. All of them were polished. On the basement of the machine, a small cylindrical mold of 24 mm diameter and 5 mm height was secured. The paste was introduced in the mold and leveled. Then the adherence test was performed in two steps. In a first step, at t = 5 min 30 s, a force of 800 g (25 kPa) was applied until the head of the mobile device was inserted into the paste by 2 mm during 20 s. In the second step, the mobile was raised at a constant rate (0.2 mm/s) and the force during the traction was monitored. The maximum tensile stress (Fmax/S, mN/mm^2^ or kPa) and the adhesion energy, normalized by the surface area (Area × g × v/S, J/m^2^) were extracted from the resulting force-displacement curves, for each cement paste and with different probes. 

### 3.5. Gentamicine release

Gentamicine sulfate (50.3 mg, 1.7%) and SE16P (56.3 mg, 2%) were added to the cement powder (2.0 g) and crushed together for two minutes. The powder was further processed as described above. A sphere was made with the cement paste and was poured at t = 5.30 min in 6 mL of pure water. The solution was stirred by hand before sampling. Samples of 50 μL of the solution were taken off at selected times and diluted to 1 mL with the HPLC eluent. Gentamicine was then titrated by HPLC according to the method described in [43]. Conditions: column HyPURITY C18 150 mm × 4 mm (Thermo), detection by refractometer (Shodex, RI-101), eluent: 97% solution of trifluoroacetic acid (TFA) in H_2_O 48.5 mM/3% MeOH, 0.7 mL/min, 20 µL injected, all species are eluted within ten minutes. Calibration with gentamicine solutions was made from 0.05 mg/ml to 2.5 mg/mL and calculated for each peak (four peaks corresponding to five species). Quantification was extracted for each peak and the mean value over the four peaks calculated, then expressed in % of the whole gentamicine included in the cement. 

### 3.6. Osteoblast culture experiments

For cell culture studies, cements tablets were prepared by using the same conditions as for injectability measurements. The selected amount of sugar derivative was mixed as usual with the cement powder. The cement paste was distributed in cylindrical molds made of teflon with a height of 2 mm and 12 mm diameter. After setting (1 hour, except for ONS10, 2 hours) the tablets were removed easily from the molds and poured in culture medium (3 mL, MEM 1X liquid with Earle’s salt and L-glutamine). The whole solution with the tablet was then sterilized by gamma radiation and incubated for seven days at 37 °C. Samples were made in triplicates. Before seeding the cells, the culture medium was removed and replaced with a fresh one (2 mL, MEM with Earle’s salt, 10% fetal bovine serum, 1% penicillin/streptomycin and 1% of a non essential amino acids solution). The medium was removed and MG63 cells, derived from human osteosarcoma, were seeded onto the samples. The seeding was performed by addition of 20 µL of medium containing 40,000 cells on the surface of the tablets and incubation of the samples for 1 hour at 37 °C under a humidified atmosphere of 95% air and 5% CO_2_. After this pre-adhesion period, 1.5 mL of culture medium was added to the wells (wells with tablets and control Tissue Culture Plastic (TCP) wells without tablets), and the samples were incubated at 37 °C for 24 hours. The cell viability was then quantified by the colorimetric MTT assay. (Briefly, after removal of the medium, the tablets were washed twice with PBS and incubated in 1 mL of medium containing 0.5 mg/mL of MTT [3-(4,5-dimethylthiazol-2-yl)-2,5-diphenyltetrazolium bromide] at 37 °C for 3 hours. The tablets are finally immersed into dimethylsulfoxide and crushed to dissolve the purple formazan crystals produced by the viable cells. After centrifugation, the absorbance of the solution is measured at 570 and 650 nm. The difference of absorbance obtained (570–650 nm) is proportional to the number of viable cells on the samples). The number of viable cells was expressed as percent relative to the number of viable cells in the control TCP well. The morphology of the osteoblasts cultured on the tablets for 24 h was observed by SEM. After washing twice with PBS, cells were fixed in a solution of glutaraldehyde (2%) in cacodylate buffer (0.1 M) and stored at 4 °C. Cells were dehydrated through a series of increasing concentrations of ethanol from 30 to 100%. The ethanol was replaced by CO2sc, and the latter was removed over its critical point. The tablets were finally coated with Au-Pd and observed with a Scanning Electron Microscope Hitachi S450 (CMEAB, Toulouse). Both the surfaces of the tablets and the sections were observed. Sections were observed in order to assess coarsely the penetration depth of the cells inside the tablets. For this purpose, the cross sections were observed from very low magnification to high magnification. At low magnification, the depth level was assessed since the whole section could be observed. Then, zooming to different parts of this cross section was performed, deeper and deeper, in order to assess the presence of cells.

## 4. Conclusions

In this study, we have shown that the addition of sugar surfactants greatly improved the injectability of calcium phosphate cement pastes, some of the additives allowing very easy extrusion of the whole sample. Otherwise, sugar surfactants brought adhesiveness to the cement pastes, which was more pronounced with the most hydrophilic sucrose esters. More insight has shown that the sucrose ester SE16P (sucrose palmitate), displaying balanced hydrophile-lipophile properties, displayed best results in terms of injectability, tackiness and porosity increase. Optimization of the formulation should lead to the selection of an amount between 1 and 2% of SE16P. From the point of view of biocompatibility, SE16P showed good results, since it allowed good osteoblast viability and anchorage with 1% surfactant.

## Supplementary Materials

Supplementary File 1
