# Supplementary material for: Calcium Phosphate Bone Cements Including Sugar Surfactants: Part Two—Injectability, Adhesive Properties and Biocompatibility
Source: Materials (Basel). 2010 Dec 2;3(12):5111–29. doi: 10.3390/ma3125111 (PMC5445814; doi:10.3390/ma3125111)
Supplement: Supplementary File 1 [file materials-03-05111-s001.pdf]

Supplementary Materials

S1: Chemical structure of sugar surfactants.

General structure of sucrose fatty acid esters.

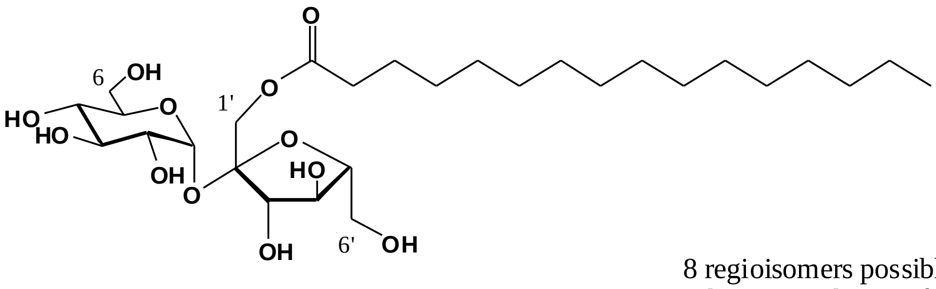

Sucrose fatty acid esters (sucroesters, SE)

8 regioisomers possibles and
substitution degrees from 1 to 5 or more

Represented here: 1'-O-palmitoylsucrose

General structure of alkylpolyglucosides.

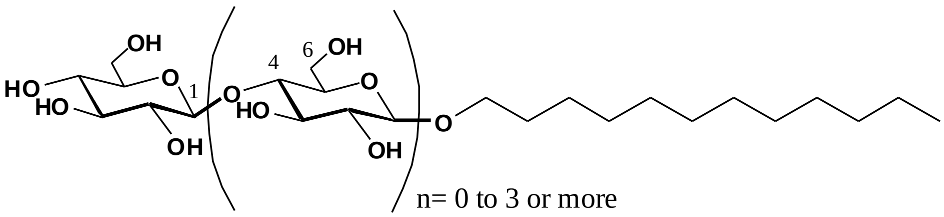

alkylpolyglucosides (APG)

anomeric configuration: α or β
glycosidic bond configuration: α or β, 1,4 or 1,6

Represented here: dodecyl(poly)glucoside with β1,4 bond

S2: Home-Made Devices.

Injection device.

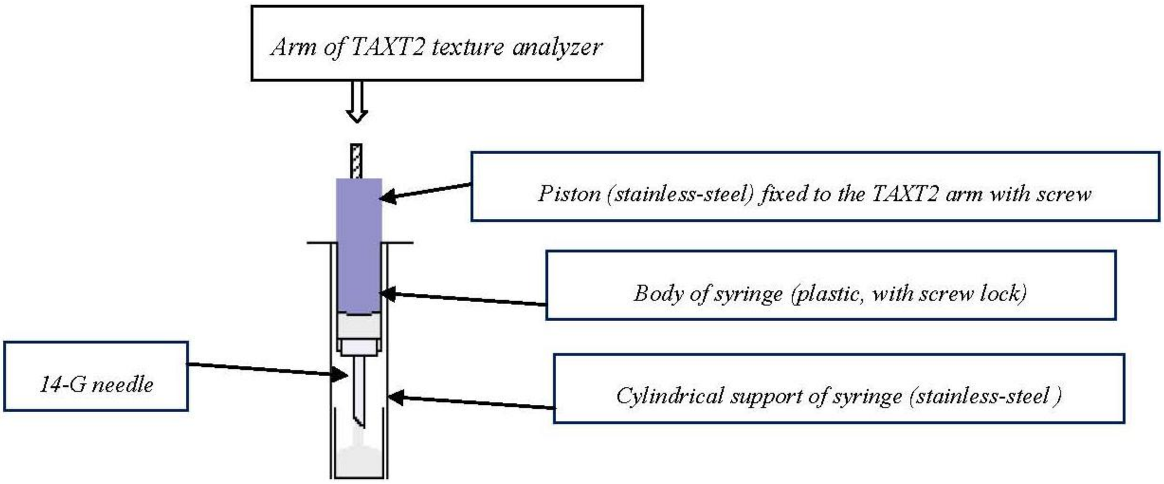

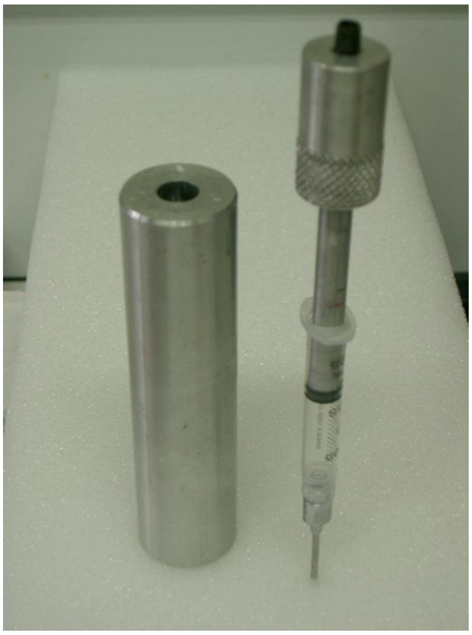

Probe-tack measurement device.

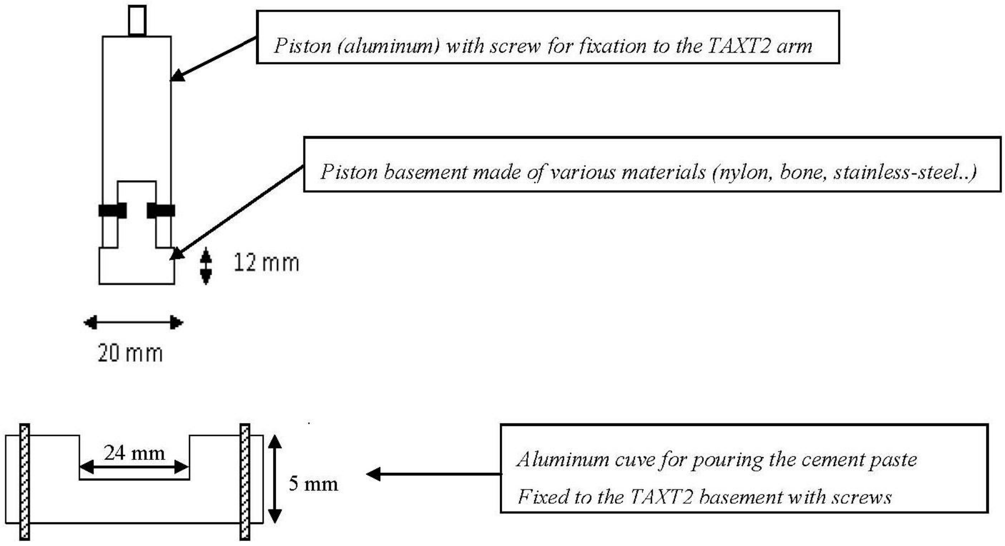

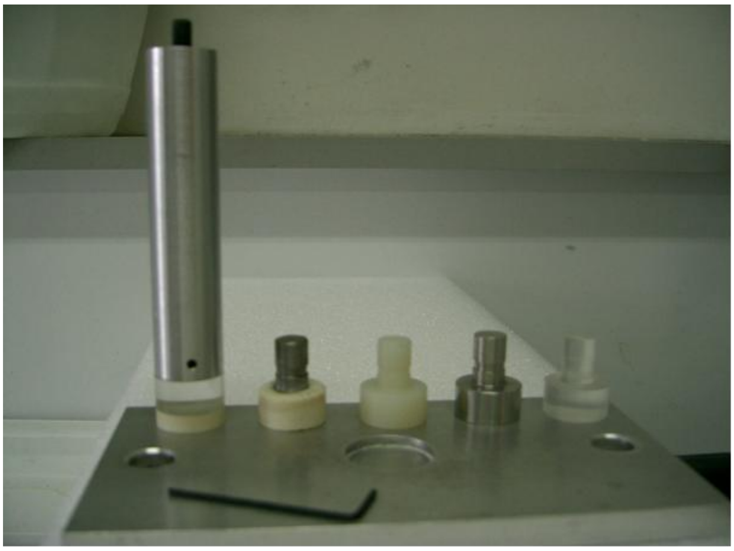

Principle of Probe-Tack measurements.

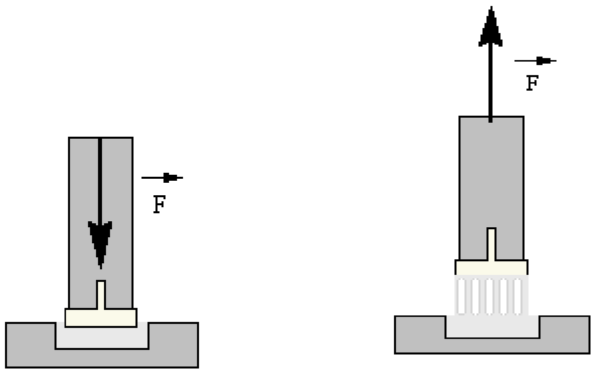

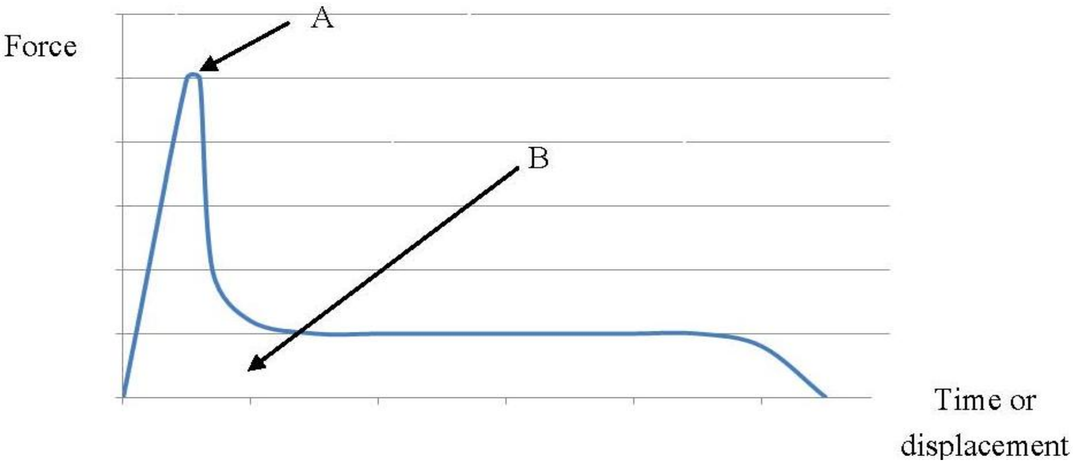

A = Adhesion strength (peak max); B = Adhesion energy (surface)

Molds for the preparation of cylinders for compressive strength measurements.

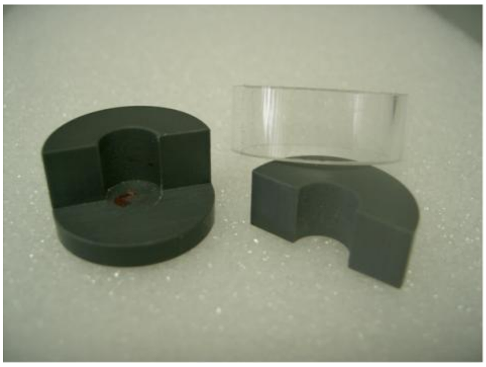

S3: Adhesion energies of cement pastes with stainless-steel probe.

With cementek.

Adhesion energy (J/m^2)	Nylon	Stainless-steel	Bone
0%	0.6	0.65	0.4
**SE16P 1%**	**2.0**		
**SE16P 3%**	**2.4**		
**SE16P 5%**	**9.3**	**4.6**	**6.5**
SE16P 10%	3.4	2.9	**7.4**
SE16P 20%	2.8		
SE16L 5%	1.7	0.8	1.4
SE16L 10%	-	-	4.6
SE11S 3%	2.3	-	1.2
SE11S 5%	0.5	-	2.2
SE5S 1%	0.9	-	-
SE5S 3%	1.6	-	-
SE5S 5%	1.4	-	-
SE5S 10%	1.8	-	-
M68EC 5%	1.4	0.4	1.9
M14 5%	0.9	0.7	1.1
ONS10 5%	0.08	0.9	0.01

With Cementek LV/sugar surfactants at 5%.

		Adhesion energy (J/m^2)
Nylon	SE16P	**3.8**
	SE11S	0.8
	SE16L	1.4
	Montanov 68EC	1.4
	Montanov 14	1.3
	No surfactant	0.5
Stainless-steel	SE16P	**3.5**
	SE11S	0.8
	No surfactant	0.9
Bone	SE16P	**2.0**
	SE11S	0.7
	SE16L	1.8
	Montanov 68EC	0.4
	Montanov 14	0.9
	No surfactant	0.4

S4: Surfactant release from cement tablets.

Surfactant release: experimental. Tablets have been prepared as described above. They have been weighted just after setting. They have been poured in 2 mL of an aqueous solution of sodium azide (0.02 wt %, 20 mg NaN_3 in 100 ml H_2O) (Analysis of release in the Earle's culture medium could not have been made because of superposition of some components of this medium on the HPLC chromatograms). After 24 h, 50 µL has been taken off and diluted with 200 µL of eluent. The diluted aliquot is analysed by HPLC. Conditions: SE16P, SE11S, M14 and M68EC have been analysed with: Column C8-grafted, MeOH/H2O 84/16 + 0.5 ml AcOH/L, 0.8 mL/ min, 20 µL injected. Main peaks: SE16P and SE11S: 7.5 min (double peak, monopalmitic sucrose esters) and 9.3 min (double peak, monostearic sucrose esters), 12.9 min (palmitic acid), 18.2 (stearic acid). M14: 7.3, 10.6 and 14.5 min. M68EC: 9.5, 10.7, 12.7, 14.7 and 20.9 min. ONS10 has been analysed with a different eluent: MeOH/H2O 75/25 + 0.5 mL AcOH/L: small peaks: 5.8, 6.1, 6.6, 10.7 and 17 min. Main peak: 7.3 min. In the case of SE16P, after one week, the solution have been taken off and evaporated to dryness. The residue has been diluted in 500 µL of eluent and analysed.

Surfactant release: results. The surfactants released in the culture medium have been monitored by HPLC. We estimated that 0.1 mg/ml of surfactant released can be quantified (concentration before dilution) and that trace amounts lower this concentration should be still detected. Quite surprisingly, we found that only ONS10 has been released, in a magnitude of 0.79 mg/mL after 24 h and 0.82 mg/ml after one week. For SE16P, SE11S, M14 and M68EC, even no trace amount of surfactant have been detected in the medium. This result was attributed to the relatively low solubility of these surfactants in water compared with ONS10 (and gentamicine too). In the case of the most hydrophilic one, SE16P, the final medium after one week has been concentrated to dryness in order to concentrate possible traces of surfactant, but again no surfactant neither its possible degradation products (palmitic and stearic acid) have been detected. Consequently, the effect of a possible surfactant release in the medium should not be significant, first because of these results, showing the absence of release in quite all the cases. And secondly because the initial culture medium in which tablets are poured after setting is replaced by fresh one, nearly one week after preparation and just before osteoblasts seeding.
